# Investigating the Effects of Nanogels in Promoting Protein Crystallization

**DOI:** 10.3390/ijms27093879

**Published:** 2026-04-27

**Authors:** Lata Govada, Beijia Wang, Yanmin Li, Emmanuel Saridakis, Naomi E. Chayen

**Affiliations:** 1 Division of Systems Medicine, Department of Metabolism, Digestion and Reproduction, Faculty of Medicine, Imperial College London, London W12 0NN, UK; l.govada@imperial.ac.uk (L.G.); b.wang18@imperial.ac.uk (B.W.); yanmin.li@nhs.net (Y.L.); 2 Institute of Nanoscience & Nanotechnology, NCSR “Demokritos”, 15310 Athens, Greece; e.saridakis@inn.demokritos.gr

**Keywords:** macromolecular crystals, nanogels, nucleation, crystallization phase diagram, nucleating agents

## Abstract

X-ray crystallography is still the most widely used and versatile method for structural studies of biological macromolecules. This study concerns the application of nanogels to facilitate protein crystallization, a prerequisite for X-ray crystallography. Nanogels (NGs) are nano-sized, highly crosslinked polymeric particles that have been extensively studied for chemical catalysis and drug delivery but not for protein crystal nucleation. The efficacy of six types of nanogels (three N-isopropylacrylamide-based and three acrylamide-based) was tested, with promising results. They were subsequently functionalised with active hydroxyl groups for further testing. Both functionalised and non-functionalised nanogels were tested on model (trypsin, thaumatin, proteinase K, ferritin and catalase) and target proteins (glulisine, α-crustacyanin and acriflavine resistance protein subunit AcrB) using both manual and automated techniques. All nanogels were found to be effective in promoting protein crystallization in both screening and optimization trials, giving crystal ‘hits’ that would have otherwise been missed. Overall, the functionalised nanogels were more effective. Nanogel effects are proposed to be due to a combination of surface porosity and surface chemistry.

## 1. Introduction

X-ray crystallography offers the most versatile and widely used method for protein structure determination. However, it requires the availability of diffracting-quality crystals, which continues to be a rate-limiting step [1]. One way to obtain high-quality crystals is to control the nucleation stage, which is the first step that crucially influences the entire crystallization process. Inducing and controlling nucleation has therefore been a key topic of research in the field for many years. Crystals can only grow from a nucleus, so it is important to encourage nucleation while preventing excess formation, where a shower of microcrystals will form as a result. A promising method is to introduce a heterologous material that possesses nucleation-inducing properties into crystallization trials [1,2,3]. These materials, which can promote nucleation and therefore crystal formation, are called nucleating agents or nucleants ([1,2,3] and references therein).

Nucleants promote nucleation in the so-called metastable zone of conditions, where no spontaneous nucleation occurs. The metastable zone conditions are considered to be the optimum conditions for crystal growth, as they allow slower crystal growth without the production of further nuclei [4]. The interest of using nucleants is thus two-fold: to promote nucleation at known metastable conditions in order to produce higher-quality crystals and to ‘reveal’ screening conditions that happen to be metastable for the specific protein, which would have remained clear if no nucleant had been introduced in the trial.

Porous materials, including hydrogels [5,6,7,8], as well as (porous and nonporous) micro- and nanoparticles (see below), have been quite successful at promoting heterogenous nucleation and influencing protein crystallization in various ways. The first generation of porous nucleants was solids: porous silicon [9,10], porous glass [11], bioglass [12], gold nanoparticles [13], porous microspheres [14] and 3D nanotemplates [15]. However, the pore sizes and/or surface chemistry of these early nucleants are difficult to control [16], and their introduction into crystallization trials is impractical and time-consuming. The next generation of nucleants was semi-liquids, including molecularly imprinted polymers (MIPs) [3], polyethylene glycol (PEG)-based hydrogels [17], graphene-based semi-liquid nucleants [1] and metal–organic frameworks [18]. MIPs were shown to be compatible with automated crystallization trials, where nanovolume crystallization drops are dispensed by liquid-handling robots [19].

There is an ongoing search for more nucleants that lend themselves to automation. Nanogels (NG) are possible candidates for which the surface porosity and chemistry can be exploited. Nanogels are nanoscale colloidal systems consisting of a hydrophilic polymeric network with surface porosity [20,21,22,23]. They have very similar gel structures and properties to standard hydrogels, except that they present themselves as nanosized spherical particles instead of macroscopic continuous gels. They therefore combine the properties of hydrogels with those of nanoparticles [24]. Hydrogels, but not nanogels, have already been shown to influence crystallization in a variety of ways, including in promoting or on the contrary inhibiting nucleation [5,25].

Due to their small sizes (both in terms of particle and mesh/pore sizes), nanogels have a large surface-area-to-volume ratio, making them beneficial for surface interactions with protein molecules in solutions [26]. One benefit of nanogels (and hydrogels in general) for crystallization trials is their tuneable characteristics, which allows them to exhibit different surface chemistries depending on the choice of monomer and on functionalization. Specific chemical interactions, such as hydrogen bonding, can contribute to stronger binding of protein molecules and thus further aid crystallization [27,28]. Their softness and ability to respond to external stimuli such as temperature, pH and ionic strength make nanogels/hydrogels interesting soft materials for a broad range of applications, including biological applications [29].

## 2. Results

Two types of trials were conducted. The first type, labelled here as “manual trials”, involved setting crystallization drops with and without nanogels (the latter being the controls) at conditions previously identified as metastable or at any rate near-metastable in order to assess whether nanogels could induce nucleation at conditions where controls remained clear or whether they could reduce the nucleation induction time. The presence of nanogels may influence crystallization, including its crystal growth stage, in more than one way, as is well known for other types of hydrogels [8]. However, a comparison in the times of appearance of the first visible crystals (or appearance of crystals versus a clear drop within a reasonable time scale which we fixed to 4 weeks) was considered an indication of differences in the nucleation induction time (and of whether spontaneous nucleation is at all possible under the given conditions) rather than growth effects. Crystallization conditions were thus first ascertained for each protein (requiring the adjustment of known conditions due to imperfect reproducibility), and the supersaturation was subsequently lowered to reach metastability. These manual trials were dispensed using the hanging-drop vapour diffusion (VD) and microbatch techniques, as detailed below, and included five proteins. The second type, labelled here as “automated screening trials”, involved screening the proteins against a large number of pre-mixed commercially available conditions. These trials were dispensed with a crystallization robot and included five proteins, three of which were in common with the manual trials. After these trials, a target membrane protein, acriflavine resistance protein subunit AcrB, was tested, both manually and with a customized grid of screening conditions.

### 2.1. Manual Testing via Vapour Diffusion and Microbatch Techniques

Nanogels prepared with N-isopropylacrylamide (NIPAM) and acrylamide (AAm) (three each; see the Materials and Methods Section) and a further five hydroxyl functionalised nanogels (NGOHs; see Section 4) were initially tested on five proteins manually, using both the hanging-drop vapour diffusion (VD) and microbatch methods. Table 1 and Table 2 summarize the main results. Negative results (clear cages) correspond to drops that remained clear for at least 4 weeks (the time limit of our observations). The results for each protein are shown below.

#### 2.1.1. Glulisine

NIPAM-based NGs and NGOHs were able to produce crystals after 1 day for both the VD and the microbatch trials. The AAm-based NGs also produced crystals after 1 day only when using the VD method, while the AAm-based NGOHs gave crystals after 1 day for both methods. Control drops in the VD trials remained clear for 4 weeks (the time limit of our observations), while the control drops in the microbatch produced crystals after 6 days.

#### 2.1.2. Thaumatin

With the VD method and the protein concentration at 20 mg/mL, all NGs and AAm-based NGOHs produced crystals after 1 day. The NIPAM-based NGOHs produced crystals after 2–3 days. The controls yielded crystals after 7 days.

At a lower protein concentration of 15 mg/mL, crystals appeared after between 3 and 6 days for both types of NGs and the NIPAM-based NGOHs. The AAm-based NGOHs were more effective, producing crystals after 1 day. The controls in this case produced crystals only on day 16.

The nanogels were ineffective with the microbatch method at all protein concentrations.

#### 2.1.3. Trypsin

With the VD method and the protein concentration at 60 mg/mL, both types of NGs gave crystals after 7–10 days, while the NIPAM-based NGOHs gave crystals within 2 days, and the controls remained clear. No crystals appeared in the microbatch.

At the lower concentration of 40 mg/mL, both types of NGs and NIPAM-based NGOHs were effective when using the VD method, giving crystals after 6–13 days. The AAm-based NGOHs and controls did not yield any crystals. No further microbatch experiments were performed.

#### 2.1.4. Ferritin

With the VD method, 10 mg/mL protein and 30 mM cadmium sulfate, a NIPAM-based NG gave crystals after 10 days, while a NIPAM-based NGOH gave crystals after 7 days. All the AAm-based NGs gave crystals only after 19 days, while an AAm-based NGOH gave crystals after 6 days. The controls did not yield crystals.

With the microbatch method and the protein concentration at 20 mg/mL, only a NIPAM-based NG gave crystals after 19 days, while all trials with the other NGs and NGOHs remained clear. Control drops precipitated after 1 day, with no crystals formed.

#### 2.1.5. Proteinase K

Using the VD method, with the protein at concentrations of 2.5 and 5 mg/mL, and 0.8 M ammonium sulfate, only the NIPAM-based NGOHs gave crystals after 1 day, while the trials with the NGs, the AAm-based NGOHs and controls remained clear.

With the microbatch method, at 20 mg/mL protein and 1 M ammonium sulfate, both types of NGs and NGOHs gave crystals after 1–4 days, while the controls remained clear.

### 2.2. Automated Screening with Functionalised Nanogels (NGOHs)

From our manual trials, we noted that the NIPAM-based NGOHs were more successful than the other types of nanogels (since they had a positive effect on all five proteins). These NGOHs were then screened on two proteins (trypsin and thaumatin) using customized non-PEG conditions from three commercial screens (Index and Crystal Screens 1 and 2, giving a total of 96 conditions; Tables S1–S3). PEG-containing conditions were initially excluded, as we believed that the presence of PEG might hinder the effectiveness of the nanogels. The results 4 weeks after setting up the trials are shown below.

#### 2.2.1. Trypsin

Five new hits were produced under conditions with NGOHs added but not in the control. In 13 conditions, both the control and the test drops produced hits, whereas in another four conditions, only the control produced hits.

#### 2.2.2. Thaumatin

Three new hits were produced only under conditions with NGOHs, while the control remained clear. Hits were found under four conditions in both the control and the test drop, whereas another condition only produced crystals in the control.

In order to verify the effect of PEG, trypsin trials were run again with the full Index screen (96 conditions). The results are shown below.

#### 2.2.3. Trypsin (Only Index Screen, All the Conditions)

Nine new hits were produced under conditions with NGOHs added but not in the control. In eight conditions, both the control and the test drops produced hits, whereas under another six conditions, only the control produced hits.

Some of the nanogel hits were produced in PEG-containing conditions, and it was therefore decided not to exclude PEG conditions in subsequent experiments. The same NGOHs were then screened on two more proteins (alpha-crystacyanin and catalase) using four commercial full screens (Index, Crystals 1 and 2, and PGA screens, totalling 288 conditions). The results 4 weeks after setting up the trials are shown below.

#### 2.2.4. Alpha-Crustacyanin

At 8 mg/mL protein, six new hits were produced with the addition of NGOHs. Hits were found under 18 conditions in both the control and the test drop, and only three conditions gave crystals in the control.

**Figure 1:**
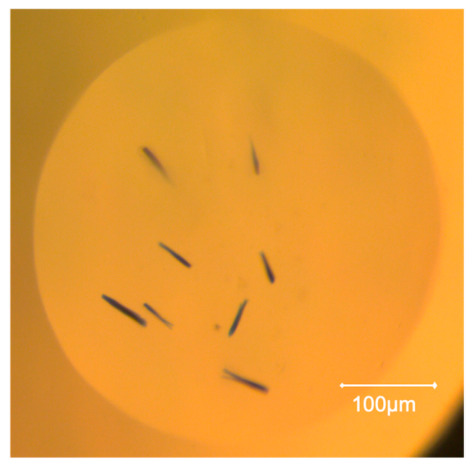
Alpha-C crystals grown in the presence of NIPAM-NGOH: 5 mg/mL α-crustacyanin with Index screen condition 62, giving larger crystals.

At a lower protein concentration of 5 mg/mL, there were nine new crystal hits with NGOHs. Crystals were found under five conditions in both the control and the test drop, whereas only two conditions yielded crystals in the controls. Larger crystals (thin rods as compared to needle-shaped crystals) were obtained under five of the conditions with NGOHs (Figure 1), compared to the controls. The difference in morphology of these crystals may be due to other factors (related to gel–protein interactions or to suppression of convective flows [30,31]), rather than or in addition to nucleating and growing in the metastable zone.

#### 2.2.5. Catalase

Four new crystal hits were obtained with NGOHs. Nine conditions yielded crystals in both the control and the test drop, whereas one condition only gave crystals in the control.

The nanogel was therefore successful in producing hits under several conditions that would not normally give crystals. The most likely explanation is that these conditions are in the metastable zone of the respective phase diagram and therefore need a heterogeneous nucleant in order to fulfil their potential as crystallization conditions.

In all the above condition screening trials, as is usual in such stochastic crystallization screenings, it cannot be excluded that a few of the ‘hits’ correspond to non-protein crystalline materials, such as the salt, buffer, or even crystals of the gels or their precursors [32,33].

### 2.3. Crystallization Trials with Membrane Protein AcrB

**Figure 2:**
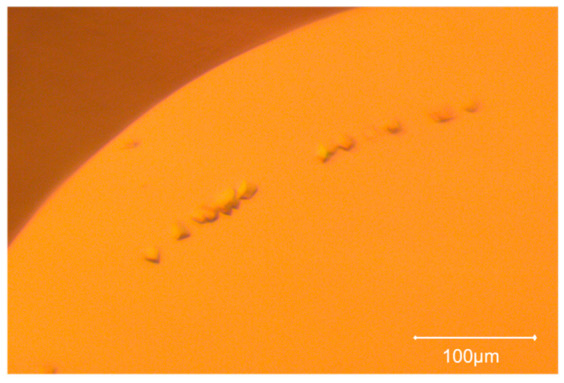
AcrB crystals grown in the microbatch in the presence of NIPAM-NGOH.

In the last stage of the experiments, the same NGOHs were tested on membrane protein AcrB using a customized systematic grid of conditions (detailed in Supplementary Information). The addition of NGOHs resulted in six more hits after 21 days (Figure 2), whereas 13 hits were common for both the control and the test drop, and only one condition gave crystals in the control.

**Figure 3:**
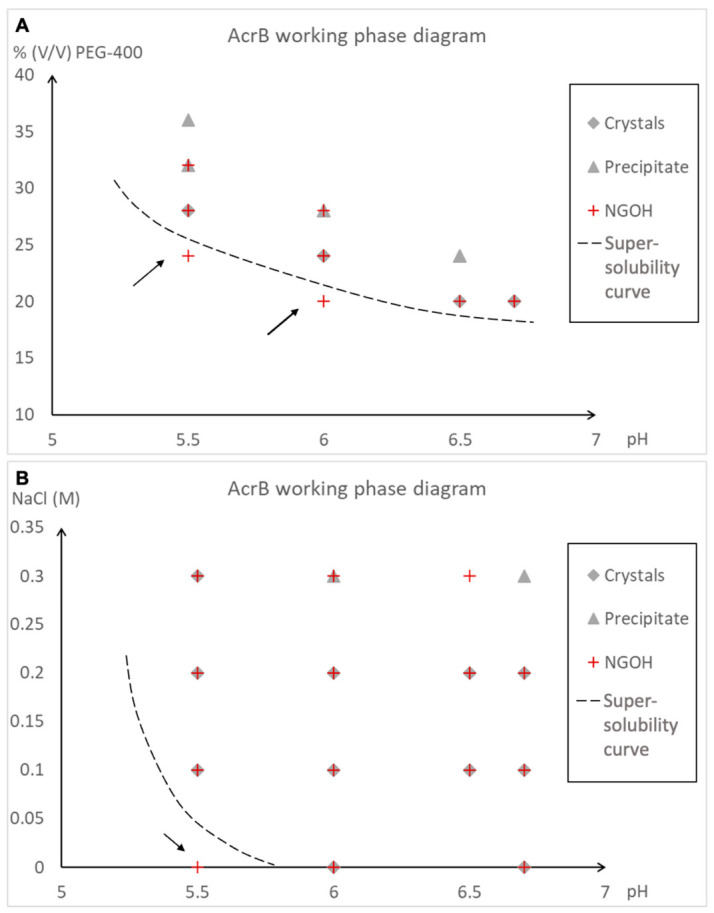
Working phase diagrams for AcrB. (**A**) Varying the %(*v*/*v*) PEG-400 against the pH; (**B**) varying the concentration of NaCl against the pH. The dotted lines represent the supersolubility curves. Grey diamonds and triangles represent the conditions that gave crystals and the precipitate, respectively, in the control trial. The red pluses indicate the conditions giving crystals in the NGOH trial. The conditions in the metastable zone where NGOHs induced crystal formation are indicated by black arrows.

A phase diagram is a representation of the distinct phases that occur and coexist at equilibrium under varying conditions [34]. For practical purposes, a working phase diagram experimentally determines the supersolubility curve, which separates the metastable zone from the nucleation zone of the diagram [4]. Two working phase diagrams (Figure 3) for AcrB at 1.5 mg/mL were produced, with two parameters varying for each diagram: one varying the PEG-400 concentration and pH and the other varying the NaCl concentration and pH. The diagrams showed that the addition of NGOHs resulted in crystal formation at metastable conditions just below the supersolubility curve, where the control drops remained clear for at least two weeks.

## 3. Discussion

As the low success rates of protein crystallization have been the ‘bottleneck’ for protein structural determination using X-ray crystallography, numerous efforts were dedicated over the past decades to understanding the crystallization processes and to looking for ways to improve crystal formation. One possible avenue is the insertion of heterogenous nucleants, which can promote the essential nucleation step of protein crystallization. To our knowledge, while nanogels have been extensively investigated for applications such as catalysis and drug delivery [35], their potential use for protein crystallization has not been studied, although standard hydrogels, including macromolecular (polymeric) and supramolecular gels, have indeed been tested and have shown promising results (e.g., [6,8,36]). The current study is the first to investigate and report the effectiveness of nanogels (i.e., hydrogels prepared as discrete nanosized particles) in promoting protein crystallization. In this study, all tested types of nanogels have shown effects in promoting crystallization of glulisine, thaumatin, trypsin, ferritin, proteinase K, alpha-crustacyanin, catalase and membrane protein AcrB to varying degrees. The nanogel was successful in producing hits under some conditions that would not normally give crystals, both under fine-tuned optimized conditions and in the sparse matrix, as well as in systematic screens. The NIPAM-NGOH, which was the most promising nanogel, could induce crystal formation in almost all cases.

Although nanogels are in fact a special category of hydrogels (they can be described as hydrogel nanoparticles), there are important practical differences. Firstly, as noted in Section 1, they have the properties of nanoparticles in addition to those of hydrogels. Nanoparticles have proven to be successful nucleants in their own right due to their high surface-to-volume ratios (large adsorption surface areas) and curvature effects, effectively acting as ‘nucleation cores’ around which protein molecules ‘condense’ [13,37,38]. Secondly, the nanogels are dispensed as a suspension into a non-gelled crystallization mixture at a rather low volume ratio, rather than being a continuous macroscopic medium inside which the crystals will grow, as in the case of standard hydrogels. Nanogels are thus used as additives rather than as a crystal growth medium, as typically done for ordinary hydrogels. In contrast to the latter, they can therefore be expected to influence the nucleation stage more than the crystal growth one.

Nanogels exerted a certain degree of crystallization-promoting effects regardless of the molecular sizes or pI of the proteins or the pH of the crystallization condition. There must therefore be a common mechanism facilitating crystallization for all nanogels. As shown in previous studies on NIPAM-based nanogels, the nanogels appeared to form spherical particles consisting of a mesh network when observed under transmission electron microscopy (TEM) [39]. Hence, one common property for all nanogels is the porous mesh network. This is consistent with previous reports of porous materials [3,9,11,12,14,40,41] and in particular hydrogels ([8] and references therein) successfully promoting protein nucleation and crystallization.

In addition to the porous network structure, another property that all nanogels have in common is a surface chemistry that allows them to attract protein molecules on their surfaces. The adsorption of protein molecules can lead to localized supersaturation on the surface of nanogel structures, which is then conducive to spontaneous nucleation [14]. Hydrogen bonding may be a key contributor to attracting protein molecules. In this respect, parallels can be drawn with protein crystallization in ionic-liquid hydrogels combined with hydrophobic membranes (IL-HCMs), where protein crystals grow completely immersed in the IL hydrogel layer possibly due in part to hydrogen bonding with the protein [42] or to protein crystallization in deep eutectic solvents (DESs) where, again, DES–protein–water interactions are crucial [43]. The difference between the two types of nanogels that were used in this study was the addition of the co-monomers NHEA and NHMA in the NGOHs.

One other important factor that differs between NGOHs and NGs is the rigidity of the mesh structure. As the additional hydrogen bonding increases the degree of cross-linking within the NGOH particles, the rigidity of the particles is much higher in NGOHs than in NGs [44]. Higher rigidity means that the nanogels are less affected (i.e., less likely to expand or shrink) [45] by various crystallization conditions. The combination of these last two factors may explain why NIPAM-NGOH was effective over the widest range of proteins. The salt in the crystallization condition may also impact the size and conformation of nanogels [44], adding an extra parameter that would make them more effective in the presence of the crystallization condition of certain salts rather than others.

The characteristics of the nanogels can vary depending on the type and percentage of monomers, initiators and cross-linkers used in the nanogel polymerization preparations. For example, depending on the monomer chains used, nanogels may have a more random distribution of mesh structures/pore sizes [46], which may contribute to successful crystallization for a wider range of proteins with different sizes [12]. These differences could also explain why certain types of nanogels were more effective for most or all proteins but would require a larger and systematically varied pool of nanogel preparation conditions in order to bring to light and rationalize such effects.

The absorption of protein molecules by the surfaces of nanogels is thought to be predominantly governed by electrostatic interactions. Higher electrostatic attraction would lead to more accumulation of protein molecules, resulting in higher chances of inducing nucleation. The nanogels prepared with a negatively charged agent, AMPS or AAc, have an overall negative charge. However, there is no evidence in our results suggesting that nanogels preferentially attracted molecules with an opposite overall charge (as determined by the value of pH-pI), which likely indicates that local electrostatic attractive interactions dominate over repulsive effects due to the overall protein charge or that some other effects prevail.

In the manual trials, the nanogels functioned better with the vapour diffusion technique. In this method, equilibration between the crystallization drop and the mother liquor is slowly achieved. The reduced ability to induce nucleation on the nanogels when using the microbatch method may possibly be caused by their amphiphilic properties [47], where the hydrophobic groups of the nanogels can possibly interact with and re-orientate towards the paraffin oil, resulting in altered nanogel conformation and reduced effectiveness.

The added volume of nanogels in the trials was not compensated in the controls by an additional volume of buffer in order to equalize the reagents’ concentrations. This means that the concentration of the protein and precipitating agents was always slightly lower in the nanogel trials than in the corresponding controls. This factor may explain the presence of crystals in some of the controls, while the corresponding nanogel trial did not yield any crystals. It is likely that, if the concentrations were equal in the test and control drops, the positive effect of the nanogels would become more obvious. Other factors preventing crystallization in the presence of nanogels at specific conditions are of course also likely to be involved.

Since the choice of the types and amount of monomers and crosslinkers can allow the tailoring of both the chemical structure and surface chemical properties of nanogels, it is important to understand how the nanogel properties influence their crystallization-promoting effect. For example, it has consistently been reported that hydrogel fibres might directly interact with protein molecules, leading to protein–gel composite crystals [8,30]. Therefore, nanogels should be further characterized in order to allow better understanding of the individual effects of each nanogel at facilitating protein crystallization. This can potentially allow the design of specific nanogels with desired properties for crystallizing proteins of interest, as well as more generic ones that would on the contrary be more suited for as wide a range of proteins as possible.

The overriding consideration is however to optimize nanogels that induce nucleation as ‘deep’ as possible into the metastable zone, i.e., as far below the supersolubility curve as possible. This is likely to lead not only to crystals appearing faster or at additional screening conditions but also to crystals being larger and of higher diffraction quality, which is of course the most important contribution of a successful nucleant.

## 4. Materials and Methods

### 4.1. Proteins

The following proteins were used for the crystallization trials. The stated concentrations were retained after preliminary testing centred around published conditions (see Section 4.2 below):

- Glulisine from the commercial insulin preparation Apidra (5F337A, Sanofi S.A., Paris, France) at 3.49 mg/mL.
- Thaumatin from *Thaumatococcus daniellii* (T7638, Sigma-Aldrich, St. Louis, MO, USA) at 15, 20 and 32.5 mg/mL in double distilled H_2_O.
- Trypsin from bovine pancreas (T9201, Sigma-Aldrich, USA) in 10 mM calcium chloride, 10 mg/mL benzamidine hydrochloride, and 20 mM HEPES at pH 7.0 and at 40 and 60 mg/mL.
- Ferritin from the equine spleen (F4503, Sigma-Aldrich, USA) at 10 and 20 mg/mL in double distilled H_2_O.
- Proteinase K from *Tritirachium album* (P2308, Sigma-Aldrich, USA) in 10 mM HEPES at pH 7.0 and at 2.5, 5 and 20 mg/mL.
- Alpha-crustacyanin (α-C) (full length) provided by Dr Peter Zagalsky of the Royal Holloway University of London at 5 mg/mL in 0.1 M Tris and pH 6.5.
- Catalase from a bovine liver (C-9322) from Sigma-Aldrich/Merck, Gillingham, UK, at 15 mg/mL in 10 mM HEPES at pH 7.0.
- AcrB [48,49] provided by Dr Isabel Moraes from the National Physical Laboratory (NPL) at 1.5 mg/mL in 10 mM Tris, 300 mM NaCl, 0.03% n-dodecyl-β-D-maltoside (DDM), and 5% glycerol at pH 7.5.

All other chemicals were obtained from Sigma-Aldrich/Merck, Gillingham, UK.

### 4.2. Crystallization Conditions for Manual Trials

In order to determine the most suitable conditions for testing the nanogels (metastable conditions but at a not excessively low supersaturation), a range of concentrations of precipitating agents was tested, with concentrations centred around published crystallization conditions for each protein. The conditions that were retained were as follows:

- Glulisine: 0.1 M Bis-TRIS 0.3 M magnesium formate at pH 5.5 [50].
- Thaumatin: 0.55 and 0.65 M ammonium tartrate dibasic at pH 7.0.
- Trypsin: 1.6 M ammonium sulfate and 0.1 M Tris at pH 8.5.
- Ferritin: 0.8 and 1.0 M ammonium sulfate, 30 and 60 mM cadmium sulfate, and 0.1 M Tris at pH 7.5. Crystallization drops were dispensed in a 2:1 protein/precipitant ratio.
- Proteinase K: 0.1 M Tris with 0.8 M ammonium sulfate at pH 8.5.

### 4.3. Crystallization Screens for Automated Screening Trials

The Index screen (HR2-144) and Crystal Screens 1 and 2 (HR2-110) were purchased from Hampton Research, Aliso Viejo, CA, USA. The PGA screen (MD1-50) was purchased from Molecular Dimensions, Rotherham, UK. The MES grid (Figure S1) customized for the AcrB protein was provided by Dr Isabel Moraes from the National Physical Laboratory (NPL).

### 4.4. Nanogels

The first series of nanogels used in this study was prepared using either N-isopropylacrylamide (NIPAM) or acrylamide (AAm) as backbone monomers, which was covalently cross-linked with N,N’-methylenebisacrylamide (MBA). The preparation and characterization details of these families of nanogels, which were synthesized by high dilution free radical polymerisation (HDFRP), are described in Reference [47] for NIPAM and Reference [51] for AAm-based nanogels. Using HDFRP results in intramolecular growth and prevents the macrogelation of the polymer. These nanogels have particle sizes of ca. 10 nm, as ascertained by dynamic light scattering (DLS). The mesh (pore) sizes are of course variable within each material and also depend on swelling, but they can reasonably be expected to be down to about one order of magnitude smaller. Some were prepared with the co-monomer 2-acrylamido-2-methylpropane sulfonic acid (AMPS), which possesses a strong negative charge at the pH values considered here. In others, acetic acid (AAc), which has a weaker negative charge, was used (Table 3). To further functionalize the nanogels for additional surface hydrophilicity (i.e., a higher degree of hydrogen bonding), a second series of hydroxyl-functionalised nanogels was prepared, where co-monomers that contained alcohol groups were incorporated instead, namely N-hydroxymethylacrylamide (NHMA) for AAm-based nanogels and N-hydroxyethylacrylamide (NHEA) for NIPAM-based nanogels (Table 3).

Therefore, four categories of MBA cross-linked nanogels were tested: (i) using AAm as the backbone monomer (AAm-based NG); (ii) using NIPAM as the backbone monomer (NIPAM-based NG); (iii) using AAm as backbone monomer and functionalized with NHMA (AAm-based NGOH); (iv) using NIPAM as backbone monomer and functionalized with NHEA (NIPAM-based NGOH). Nanogels at concentrations of 1, 3, 5 mg/mL were tested, but no significant differences in the results were observed between these nanogels, indicating that 1 mg/mL is a sufficient concentration to produce the desired effect.

### 4.5. Manual Crystallization Trials

Hanging-drop vapour diffusion crystallization trials were set up using 24-well Linbro-style XRL plates (Molecular Dimensions, Suffolk, UK) or EasyXtal plates (Qiagen, Hilden, Germany). For each trial, the well was filled with 400 μL of a chosen precipitant (as reservoir). On a siliconized cover slip (Molecular Dimensions, Suffolk, UK) or an X-Seal crystallization support (Qiagen, Germany), 1 μL of protein was mixed with 1 μL of the corresponding precipitant (1:1 volume ratio except for ferritin at a 2:1 protein/precipitant ratio) to create a crystallization drop. Each cover slip or X-Seal support was inverted and sealed onto the well containing the same precipitant.

Microbatch experiments were performed using Nunc HLA Terasaki 72-well plates (Douglas Instruments, Hungerford, UK). The plates were cleaned with compressed air and then filled with 100% paraffin oil. Protein and crystallization solution drops were dispensed under the paraffin layer at a 1:1 ratio (except for ferritin at a 2:1 protein/precipitant ratio) and allowed to mix.

For each condition in the vapour diffusion and microbatch crystallization trials, a nucleant drop containing a 0.2 µL nanogel (i.e., 10% of the drop volume) was set up alongside a control drop containing no nucleant.

### 4.6. Automated Crystallization Trials

The Oryx-8 protein crystallization robot system fitted with the WaspRun screening software V23.96 (Douglas Instruments, UK) was used for the automated screening trials, and 96-well SwissCI MRC two-drop plates (MD-11, Molecular Dimensions, UK) were used.

The sitting-drop vapour diffusion method was used for the screening setup. Each protein was mixed with each screen condition (precipitant) in a 1:1 ratio, yielding a 400 nL drop. This was equilibrated against an 80 μL reservoir containing only the screen condition.

To test the effectiveness of the nanogels in automated screening trials, 0.02 µL or 0.04 µL (5% or 10% of drop volume) of a nanogel was dispensed into the crystallization drop for each condition. No additional effect was observed at the higher nanogel volume, so these trials were discontinued, and only results for the 0.02 µL nanogel are reported here. A negative control drop containing no nucleant was also set up for each condition.

All trials were carried out at 22 °C and observed for 4 weeks with a Leica M165 C stereoscope (Leica Microsystems, Mannheim, Germany). Images were taken with a Leica DFC295 camera and processed with Leica Application Suite V3.3.0 software (Leica Microsystems, Mannheim, Germany).

## Figures and Tables

**Table 1:** Summary of the manual vapour diffusion trials of five proteins with 1 mg/mL nanogels.

	Conc. (mg/mL)	MW	NIPAM (NG1)	NIPAM-AMPS (NG3)	NIPAM-AAc (NG5)	AAm (NG2)	AAm-AMPS (NG4)	AAm-AAc (NG6)	75%NIPAM (NGOH1)	70%NIPAM (NGOH2)	60%NIPAM (NGOH3)	50%AAm (NGOH4)	70%AAm (NGOH5)	Control
Glulisine	3.49	6												
Thaumatin	15	22												
Trypsin	40	23.8												
Ferritin	10	450												
Proteinase K	2.5	28.9												

Dark green: crystal appearance ≤ 7 days, Middle green: crystal appearance between 7 and 14 days, Light green: crystal appearance >14 days.

**Table 2:** Summary of the manual microbatch trials of five proteins with 1 mg/mL nanogels.

	Conc. (mg/mL)	MW	NIPAM (NG1)	NIPAM-AMPS (NG3)	NIPAM-AAc (NG5)	Aam (NG2)	AAm-AMPS (NG4)	AAm-AAc (NG6)	75%NIPAM (NGOH1)	70%NIPAM (NGOH2)	60%NIPAM (NGOH3)	50%AAm (NGOH4)	70%AAm (NGOH5)	Control
Glulisine	3.49	6												
Thaumatin	32.5	22												
Trypsin	60	23.8												
Ferritin	20	450												
Proteinase K	20	28.9												

Dark green: crystal appearance ≤ 7 days, Middle green: crystal appearance between 7 and 14 days, Light green: crystal appearance >14 days.

**Table 3:** Detailed compositions of the nanogels.

	Monomer 1 (Backbone)	Co-Monomer/Agent	Cross-Linker
NG1	80% NIPAM	-	20% MBA
NG3	60% NIPAM	20% AMPS	20% MBA
NG5	60% NIPAM	20% AAc	20% MBA
NG2	80% AAm	-	20% MBA
NG4	60% AAm	20% AMPS	20% MBA
NG6	60% AAm	20% AAc	20% MBA
NGOH1	75% NIPAM	20% NHEA	5% MBA
NGOH2	70% NIPAM	20% NHEA	10% MBA
NGOH3	60% NIPAM	20% NHEA	20% MBA
NGOH4	50% AAm	30% NHMA	20% MBA
NGOH5	70% AAm	10% NHMA	20% MBA

## Data Availability

The original data presented in this study are included in the article/Supplementary Material. Further inquiries can be directed to the corresponding author.

## Supplementary Materials

The following supporting information can be downloaded at https://www.mdpi.com/article/10.3390/ijms27093879/s1.

## Abbreviations

The following abbreviations are used in this manuscript:

MIPs	Molecularly imprinted polymers
VD	Vapour diffusion
AAm	Acrylamide
NIPAM	N-isopropylacrylamide
MBA	N,N’-methylenebisacrylamide
AMPS	2-acrylamido-2-methylpropane sulfonic acid
AAc	acetic acid
NG	Nanogel
NGOH	Hydroxyl functionalised nanogel
NHMA	N-hydroxymethylacrylamide
NHEA	N-hydroxyethylacrylamide
PEG	Polyethylene glycol
