# Supplementary material for: Investigating the Effects of Nanogels in Promoting Protein Crystallization
Source: Int J Mol Sci. 2026 Apr 27;27(9):3879. doi: 10.3390/ijms27093879 (PMC13163816; doi:10.3390/ijms27093879)
Supplement: Supplementary file 1 [file ijms-27-03879-s001.zip › ijms-4199696-supplementary.pdf]

Supplementary Information

Figure S1: The 96 conditions of the MES matrix for AcrB

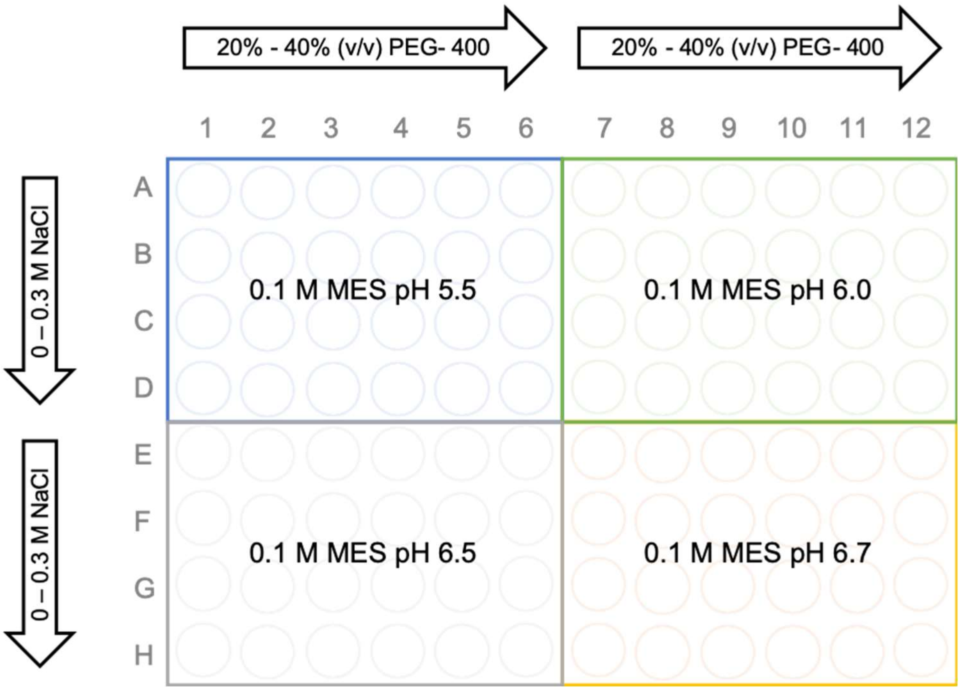

List of non-PEG conditions used for the trypsin and thaumatin screening experiments.

Table S1: Index screen formulation excluding PEG conditions

Reagent number	Contents
1	0.1 M Citric acid pH 3.5, 2.0 M Ammonium sulfate
2	0.1 M Sodium acetate trihydrate pH 4.5, 2.0 M Ammonium sulfate
3	0.1 M BIS-TRIS pH 5.5, 2.0 M Ammonium sulfate
4	0.1 M BIS-TRIS pH 6.5, 2.0 M Ammonium sulfate
5	0.1 M HEPES pH 7.5, 2.0 M Ammonium sulfate
6	0.1 M Tris pH 8.5, 2.0 M Ammonium sulfate
7	0.1 M Citric acid pH 3.5, 3.0 M Sodium chloride
8	0.1 M Sodium acetate trihydrate pH 4.5. 3.0 M Sodium chloride
9	0.1 M BIS-TRIS pH 5.5, 3.0 M Sodium chloride
10	0.1 M BIS-TRIS pH 6.5, 3.0 M Sodium chloride
11	0.1 M HEPES pH 7.5, 3.0 M Sodium chloride
12	0.1 M Tris pH 8.5, 3.0 M Sodium chloride
13	0.1 M BIS-TRIS pH 5.5, 0.3 M Magnesium formate dihydrate
14	0.1 M BIS-TRIS pH 6.5, 0.5 M Magnesium formate dihydrate
15	0.1 M HEPES pH 7.5, 0.5 M Magnesium formate dihydrate
16	0.1 M Tris pH 8.5, 0.3 M Magnesium formate dihydrate
17	1.26 M Sodium phosphate monobasic monohydrate, 0.14 M Potassium phosphate dibasic, pH 5.6
18	0.49 M Sodium phosphate monobasic monohydrate, 0.91 M Potassium phosphate dibasic, pH 6.9
19	0.056 M Sodium phosphate monobasic monohydrate, 1.344 M Potassium phosphate dibasic, pH 8.2
20	0.1 M HEPES pH 7.5, 1.4 M Sodium citrate tribasic dihydrate
21	1.8 M Ammonium citrate tribasic, pH 7.0
22	0.8 M Succinic acid pH 7.0
23	2.1 M DL-Malic acid pH 7.0

24	2.8 M Sodium acetate trihydrate pH 7.0
25	3.5 M Sodium formate pH 7.0
26	1.1 M Ammonium tartrate dibasic pH 7.0
27	2.4 M Sodium malonate pH 7.0
28	35% v/v Tacsimate TM pH 7.0
29	60% v/v Tacsimate TM pH 7.0
30	0.1 M Sodium chloride, 0.1 M BIS-TRIS pH 6.5, 1.5 M Ammonium sulfate
48	0.2 M Calcium chloride dihydrate, 0.1 M BIS-TRIS pH 5.5, 45% v/v (+/-)-2-Methyl-2,4-pentanediol
49	0.2 M Calcium chloride dihydrate, 0.1 M BIS-TRIS pH 6.5, 45% v/v (+/-)-2-Methyl-2,4-pentanediol
50	0.2 M Ammonium acetate, 0.1 M BIS-TRIS pH 5.5, 45% v/v (+/-)-2-Methyl-2,4-pentanediol
51	0.2 M Ammonium acetate, 0.1 M BIS-TRIS pH 6.5, 45% v/v (+/-)-2-Methyl-2,4-pentanediol
52	0.2 M Ammonium acetate, 0.1 M HEPES pH 7.5, 45% v/v (+/-)-2-Methyl-2,4-pentanedio
53	0.2 M Ammonium acetate, 0.1 M Tris pH 8.5, 45% v/v (+/-)-2-Methyl-2,4-pentanediol
56	0.2 M Potassium chloride, 0.05 M HEPES pH 7.5, 35% v/v Pentaerythritol propoxylate (5/4 PO/OH)
57	0.05 M Ammonium sulfate, 0.05 M BIS-TRIS pH 6.5, 30% v/v Pentaerythritol ethoxylate (15/4 EO/OH)
59	0.02 M Magnesium chloride hexahydrate, 0.1 M HEPES pH 7.5, 22% w/v Poly (acrylic acid sodium salt) 5,100
60	0.01 M Cobalt (II) chloride hexahydrate, 0.1 M Tris pH 8.5, 20% w/v Polyvinylpyrrolidone K 15

Table S2: Crystal Screen formulation excluding PEG conditions

Reagent number	Contents
1	0.02 M Calcium chloride dihydrate, 0.1 M Sodium acetate trihydrate pH 4.6, 30% v/v (+/-)-2-Methyl-2,4-pentanediol
2	0.4 M Potassium sodium tartrate tetrahydrate
3	0.4 M Ammonium phosphate monobasic
4	0.1 M TRIS hydrochloride pH 8.5, 2.0 M Ammonium sulfate
5	0.2 M Sodium citrate tribasic dihydrate, 0.1 M HEPES sodium pH 7.5, 30% v/v (+/-)-2-Methyl-2,4-pentanediol
7	0.1 M Sodium cacodylate trihydrate pH 6.5, 1.4 M Sodium acetate trihydrate
8	0.2 M Sodium citrate tribasic dihydrate, 0.1 M Sodium cacodylate trihydrate pH 6.5, 30% v/v 2-Propano
16	0.1 M HEPES sodium pH 7.5, 1.5 M Lithium sulfate monohydrate
19	0.2 M Ammonium acetate, 0.1 M TRIS hydrochloride pH 8.5, 30% v/v 2-Propanol
21	0.2 M Magnesium acetate tetrahydrate, 0.1 M Sodium cacodylate trihydrate pH 6.5, 30% v/v (+/-)-2-Methyl-2,4-pentanediol
24	0.2 M Calcium chloride dihydrate, 0.1 M Sodium acetate trihydrate pH 4.6, 20% v/v 2-Propanol
25	0.1 M Imidazole pH 6.5, 1.0 M Sodium acetate trihydrate
26	0.2 M Ammonium acetate, 0.1 M Sodium citrate tribasic dihydrate pH 5.6, 30% v/v (+/-)-2-Methyl-2,4-pentanediol
29	0.1 M HEPES sodium pH 7.5, 0.8 M Potassium sodium tartrate tetrahydrate
32	2.0 M Ammonium sulfate
33	4.0 M Sodium formate
34	0.1 M Sodium acetate trihydrate pH 4.6, 2.0 M Sodium formate

35	0.1 M HEPES sodium pH 7.5, 0.8 M Sodium phosphate monobasic monohydrate, 0.8 M Potassium phosphate monobasic
38	0.1 M HEPES sodium pH 7.5, 1.4 M Sodium citrate tribasic dihydrate
44	0.2 M Magnesium formate dihydrate
47	0.1 M Sodium acetate trihydrate pH 4.6, 2.0 M Ammonium sulfate

Table S3: Crystal Screen 2 formulation excluding PEG conditions

2	0.5 M Sodium chloride, 0.01 M Magnesium chloride hexahydrate, 0.01 M Hexadecyltrimethylammonium bromide
3	25% v/v Ethylene glyco
4	35% v/v 1,4-Dioxane
5	2.0 M Ammonium sulfate, 5% v/v 2-Propanol
6	1.0 M Imidazole pH 7.0
8	1.5 M Sodium chloride, 10% v/v Ethanol
9	0.1 M Sodium acetate trihydrate pH 4.6, 2.0 M Sodium chloride
10	0.2 M Sodium chloride, 0.1 M Sodium acetate trihydrate pH 4.6, 30% v/v (+/-)-2-Methyl-2,4-pentanedio
11	0.01 M Cobalt(II) chloride hexahydrate, 0.1 M Sodium acetate trihydrate pH 4.6, 1.0 M 1,6-Hexanedio
14	0.2 M Potassium sodium tartrate tetrahydrate, 0.1 M Sodium citrate tribasic dihydrate pH 5.6, 2.0 M Ammonium sulfate
15	0.5 M Ammonium sulfate, 0.1 M Sodium citrate tribasic dihydrate pH 5.6, 1.0 M Lithium sulfate monohydrate
16	0.5 M Sodium chloride, 0.1 M Sodium citrate tribasic dihydrate pH 5.6, 1.0 M Lithium sulfate monohydrate

17	0.1 M Sodium citrate tribasic dihydrate pH 5.6, 35% v/v tert-Butano
18	0.01 M Iron(III) chloride hexahydrate, 0.1 M Sodium citrate tribasic dihydrate pH 5.6, 10% v/v Jeffamine ® M-600 ®
19	0.1 M Sodium citrate tribasic dihydrate pH 5.6, 2.5 M 1,6-Hexanedio
20	0.1 M MES monohydrate pH 6.5, 1.6 M Magnesium sulfate heptahydrate
21	0.1 M Sodium phosphate monobasic monohydrate, 0.1 M Potassium phosphate monobasic, 0.1 M MES monohydrate pH 6.5, 2.0 M Sodium chloride
23	1.6 M Ammonium sulfate, 0.1 M MES monohydrate pH 6.5, 10% v/v 1,4-Dioxane
24	0.05 M Cesium chloride, 0.1 M MES monohydrate pH 6.5, 30% v/v Jeffamine ® M-600 ®
25	0.01 M Cobalt(II) chloride hexahydrate, 0.1 M MES monohydrate pH 6.5, 1.8 M Ammonium sulfate
28	1.6 M Sodium citrate tribasic dihydrate pH 6.5
29	0.5 M Ammonium sulfate, 0.1 M HEPES pH 7.5, 30% v/v (+/-)-2-Methyl-2,4-pentanediol
31	0.1 M HEPES pH 7.5, 20% v/v Jeffamine ® M-600 ®
32	0.1 M Sodium chloride, 0.1 M HEPES pH 7.5, 1.6 M Ammonium sulfate
33	0.1 M HEPES pH 7.5, 2.0 M Ammonium formate
34	0.05 M Cadmium sulfate hydrate, 0.1 M HEPES pH 7.5, 1.0 M Sodium acetate trihydrate
35	0.1 M HEPES pH 7.5, 70% v/v (+/-)-2-Methyl-2,4-pentanediol
36	0.1 M HEPES pH 7.5, 4.3 M Sodium chloride
39	0.2 M Magnesium chloride hexahydrate, 0.1 M Tris pH 8.5, 3.4 M 1,6-Hexanediol
40	0.1 M Tris pH 8.5, 25% v/v tert-Butano
41	0.01 M Nickel(II) chloride hexahydrate, 0.1 M Tris pH 8.5, 1.0 M Lithium sulfate monohydrate

42	1.5 M Ammonium sulfate, 0.1 M Tris pH 8.5, 12% v/v Glycerol
43	0.2 M Ammonium phosphate monobasic, 0.1 M Tris pH 8.5, 50% v/v (+/-)-2-Methyl-2,4-pentanediol
44	0.1 M Tris pH 8.5, 20% v/v Ethanol
47	0.1 M BICINE pH 9.0, 2.0 M Magnesium chloride hexahydrate
